# Gestational Age-Specific Biometric and Estimated Fetal Weight Curves in Gastroschisis: A Brazilian Multicenter Cohort Study

**DOI:** 10.3390/diagnostics16091402

**Published:** 2026-05-06

**Authors:** Karina Felippe Monezi Pontes, Liliam Cristine Rolo, Gustavo Yano Callado, Alberto Borges Peixoto, Manoel Sarno, Alex Sandro Rolland Souza, Francisco Herlânio Costa Carvalho, Antonio Braga, Edward Araujo Júnior

**Affiliations:** 1 Department of Obstetrics, Paulista School of Medicine—Federal University of São Paulo (EPM-UNIFESP), São Paulo 04023-062, Brazil; karinamonezi@hotmail.com (K.F.M.P.); liliamrolo@hotmail.com (L.C.R.); araujojred@terra.com.br (E.A.J.); 2 Faculdade Israelita de Ciências da Saúde Albert Einstein, Hospital Israelita Albert Einstein, São Paulo 05652-900, Brazil; 3 Gynecology and Obstetrics Service, Mario Palmério University Hospital—University of Uberaba (UNIUBE), Uberaba 38055-500, Brazil; albertobpeixoto@gmail.com; 4 Department of Obstetrics and Gynecology, Federal University of Triângulo Mineiro (UFTM), Uberaba 38025-440, Brazil; 5 Department of Obstetrics and Gynecology, Federal University of Bahia (UFBA), Salvador 40055-150, Brazil; sarnomanoel@gmail.com; 6 Caliper—School of Image, Salvador 41810-012, Brazil; 7 Service of Fetal Medicine, Instituto de Medicina Integral Prof. Fernando Figueira (IMIP), Recife 50070-555, Brazil; alexrolland@uol.com.br; 8 Academic Area of Gynecology and Obstetrics, Federal University of Pernambuco (UFPE), Recife 50670-420, Brazil; 9 School of Health and Life Sciences, Catholic University of Pernambuco (UNICAP), Recife 50050-410, Brazil; 10 Department of Obstetrics and Gynecology, Federal University of Ceará (UFC), Fortaleza 60430-270, Brazil; herlaniocosta@uol.com.br; 11 Department of Gynecology and Obstetrics, School of Medicine, Federal University of Rio de Janeiro (UFRJ), Rio de Janeiro 22240-003, Brazil; bragamed@yahoo.com.br; 12 Department of General and Specialized Surgery, School of Medicine and Surgery, Federal University of the State of Rio de Janeiro (UNIRIO), Rio de Janeiro 22290-240, Brazil; 13 Postgraduate Program in Applied Health Sciences, University of Vassouras (Univassouras), Vassouras 27700-000, Brazil

**Keywords:** gastroschisis, fetal growth, estimated fetal weight, ultrasound biometry, growth curves

## Abstract

**Objective**: This study aimed to describe gestational age-specific biometric and estimated fetal weight (EFW) patterns derived from a multicenter cohort of fetuses with gastroschisis and to evaluate the agreement between prenatal EFW and birth weight. **Methods**: This retrospective study included singleton pregnancies with a prenatal diagnosis of gastroschisis and at least two ultrasound evaluations between 20 and 37 weeks. Data were collected from four Brazilian tertiary centers between 2010 and 2024. Biometric parameters (biparietal diameter, head circumference [HC], abdominal circumference, and femur length) and EFW were recorded. EFW was calculated using Hadlock IV and Siemer formulas. Polynomial regression models were applied to generate gestational age-specific curves for HC, femur length (FL), and EFW. Agreement between prenatal EFW and birth weight (in cases assessed within 14 days of delivery) was analyzed using Bland–Altman plots and the concordance correlation coefficient (CCC). **Results**: A total of 116 pregnancies and 355 ultrasound tests were included. Polynomial models showed a strong association between gestational age and EFW (R^2^ = 0.837 for Siemer; R^2^ = 0.728 for Hadlock), HC (R^2^ = 0.849), and FL (R^2^ = 0.877). The 50th percentile curves for gastroschisis were consistently lower than those from standard growth charts. In the birthweight concordance analysis (*n* = 46), the Siemer formula showed low agreement (CCC = 0.55), while the Hadlock formula showed even lower concordance (CCC = 0.44), with both formulas underestimating actual birth weight. **Conclusions**: Fetuses with gastroschisis have distinct growth patterns not captured by standard references. Tailored growth curves and careful interpretation of EFW are essential to improve prenatal assessment in this population.

## 1. Introduction

Pregnancies complicated by gastroschisis are associated with increased risks of small for gestational age, preterm birth, stillbirth, and intestinal complications requiring surgical correction [1]. Gastroschisis has an estimated prevalence of approximately 2 to 5 cases per 10,000 live births worldwide, with increasing incidence reported in several regions, particularly among younger mothers [1]. Neonatal outcomes are largely determined by the degree of bowel involvement, with complex cases linked to higher morbidity, prolonged parenteral nutrition, and extended hospitalization [1]. Given these risks, accurate prenatal assessment and delivery planning in specialized centers are essential to optimize perinatal outcomes.

Fetal monitoring plays a central role in the management of pregnancies complicated by gastroschisis. Serial ultrasound is essential to monitor fetal well-being and identify markers associated with adverse outcomes, such as bowel dilation, polyhydramnios, and abnormal gastric bubbles [2,3]. These findings help distinguish simple from complex cases and guide delivery timing.

The study by Netta et al. [4] was pioneering in demonstrating that up to 61% of fetuses with gastroschisis were below the 10th percentile for gestational age, compared with 3–10% of normal fetuses, depending on the definition. However, studies such as that of Barbieri et al. have shown that, despite this downward shift in growth curves, umbilical artery Doppler findings were normal in 97.5% of cases [5], suggesting that this does not represent fetal growth restriction secondary to placental insufficiency but rather a constitutional growth pattern.

Traditional fetal growth charts and weight estimation formulas, such as those developed by Hadlock, have limited applicability in gastroschisis due to the anatomical distortion caused by herniated bowel loops [5,6]. These formulas rely heavily on abdominal circumference, which is systematically reduced in this population, independent of placental insufficiency. Consequently, many fetuses with gastroschisis are erroneously classified as small for gestational age, prompting unnecessary surveillance and preterm delivery. Such misclassification may lead to overtreatment while failing to identify those at true risk [7]. Tailored growth assessment strategies are therefore essential to distinguish constitutional smallness from pathological restriction, minimize intervention-related risks, and improve the precision of prenatal care.

Therefore, the aim of this study was to establish gestational age-specific curves for biometric parameters and estimated fetal weight (EFW) in fetuses with gastroschisis, using longitudinal ultrasound data from a multicenter Brazilian cohort, providing novel, population-specific reference standards for the Brazilian setting. Additionally, we sought to evaluate the agreement between prenatal EFW estimates and actual birth weight using two commonly applied formulas (Hadlock IV and Siemer) to assess their clinical applicability in this population. Notably, this study is the first to employ the Siemer formula for the construction of a weight curve in this population.

## 2. Methods

### 2.1. Study Design and Setting

This was a retrospective, longitudinal study including pregnant women carrying fetuses diagnosed with gastroschisis between 2010 and 2024. Data were obtained from four public university-affiliated tertiary referral centers in Brazil: Paulista School of Medicine—Federal University of São Paulo (EPM-UNIFESP), Federal University of Ceará (UFC), Federal University of Bahia (UFBA), and Professor Fernando Figueira Institute of Integral Medicine (IMIP). This study was approved (15 September 2023) by the Ethics Committee at the coordinating center of UNIFESP (CAAE. 68326023.3.1001.5505). Due to the retrospective nature of the study, a waiver of informed consent was requested. As this was a retrospective study, no formal sample size calculation was performed; all eligible cases during the study period were included to maximize sample size and representativeness.

### 2.2. Inclusion Criteria

Eligible participants were pregnant women with singleton gestations and fetuses with a confirmed ultrasound diagnosis of gastroschisis, resulting in live births. Inclusion required at least two prenatal ultrasound examinations performed between 20 and 38 weeks and 6 days of gestation, with complete biometric parameters—biparietal diameter (BPD), head circumference (HC), abdominal circumference (AC), femur length (FL), and EFW—and absence of associated structural anomalies or chronic maternal disease. This minimized confounding factors that could independently affect fetal growth. The diagnosis was based on the sonographic identification of a paraumbilical abdominal wall defect with free-floating bowel loops. Gestational age was determined using the last menstrual period in women with regular cycles; otherwise, first- or second-trimester ultrasounds were used for dating.

### 2.3. Follow-Up Protocol

Fetuses were followed according to each institution’s fetal medicine protocol. A biweekly Doppler ultrasound was performed from 28 weeks for fetuses with EFW above the 10th percentile or between the 3rd and 10th percentiles with normal amniotic fluid. Weekly assessments were scheduled for those with EFW < 3rd percentile or abnormal fluid volume or in the presence of bowel dilation. Fetal surveillance after 34 weeks included cardiotocography and the biophysical profile. Antenatal corticosteroid therapy with intramuscular betamethasone (12 mg every 24 h, two doses) was administered at 32 weeks, or earlier (30–32 weeks) in cases of suspected fetal compromise. Elective cesarean section was scheduled at 37 weeks to reduce prematurity-associated risks.

### 2.4. Ultrasound Parameters and Weight Estimation

Biometric measurements were performed according to the protocols of the International Society of Ultrasound in Obstetrics and Gynecology (ISUOG) [8] and by specialists in fetal medicine. Despite this standardization, some inter-center variability related to equipment and clinical protocols may persist. EFW was calculated using two equations: the Hadlock IV formula and the Siemer formula. The Hadlock IV equation is as follows: EFW = 1.3596 + (0.00061 × BPD × AC) + (0.424 × AC) + (1.74 × FL) + (0.0064 × HC) − (0.00386 × AC × FL) [9]. The Siemer formula is defined as EFW = −145.577 + (23.724 × FL^2^) + (1.255 × BPD^3^) + (0.001 × e^OFD^) − (0.0000406 × 10^FL^) + (1.03 × e^FL^), where ‘e’ represents Euler’s number (approximately 2.718) [10]. EFW values were plotted using the fetal growth charts proposed by Peixoto et al. [11] and Hadlock et al. [12] for comparison.

Although abdominal circumference was collected, it was not included in the construction of growth curves because it is directly influenced by the abdominal wall defect in gastroschisis and may not reliably reflect overall fetal growth.

### 2.5. Statistical Analysis

Data were transferred to Microsoft Excel 2010 (Microsoft Corp., Redmond, WA, USA) and analyzed using PASW Statistics version 18.0 (SPSS Inc., Chicago, IL, USA) and GraphPad Prism version 7.0 (GraphPad Software, San Diego, CA, USA). Normality of quantitative variables was assessed using the D’Agostino–Pearson omnibus test. Parametric variables were reported as means ± standard deviations (SD), and nonparametric data as medians with ranges.

Polynomial regression models (linear, quadratic, cubic) were fitted to generate gestational age-specific curves for EFW (Hadlock and Siemer), HC, and FL [13]. Measurement variability was assessed by modeling SD across gestational ages using linear regression. Percentile curves (5th, 50th, and 95th) were derived using the formula Percentile = Mean ± K × SD, where K is the corresponding Z-score.

The gestational age-specific curves for EFW, calculated using the Siemer and Hadlock formulas, as well as HC and FL, were subsequently compared with the growth curve of normal fetuses in a Brazilian population [11].

Biometric parameters were correlated using Pearson’s test (parametric) or Spearman’s test (nonparametric). Agreement between prenatal EFW and birth weight (when measured within 14 days of delivery) was evaluated with Bland–Altman analysis and the concordance correlation coefficient (CCC). Agreement was classified based on the relative difference scale proposed by Martins and Nastri: >50% very poor, 20–50% poor, 10–20% moderate, 5–10% good, and <5% very good [14]. A *p*-value < 0.05 was considered statistically significant.

## 3. Results

### 3.1. Study Population

Between 2010 and 2024, 116 pregnancies with gastroschisis were included. A total of 355 ultrasound examinations were performed between 20 weeks and 38 weeks + 6 days of gestation to establish reference values for HC, FL, and EFW calculated using the Siemer and Hadlock formulas. Clinical characteristics of the study population are summarized in Table 1. Missing data for some variables reflect incomplete records inherent to the retrospective multicenter design and are reported explicitly in Table 1.

### 3.2. Gestational Age-Specific Curves

**Figure 1:**
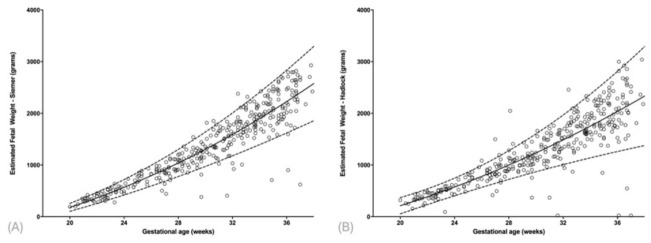
Scatter plots of estimated fetal weight (EFW) in fetuses with gastroschisis, calculated using the Siemer formula (**A**) and the Hadlock formula (**B**), as a function of gestational age.

Polynomial regression models demonstrated strong positive associations between gestational age and all biometric parameters, but the variation is large, especially after 32 weeks. For EFW estimated by the Siemer formula, a second-degree polynomial relationship was observed, with a Spearman correlation coefficient of r = 0.91 (*p* < 0.001) and a coefficient of determination (R^2^) of 0.837. This indicates that 83.7% of the variance in EFW was explained by gestational age. On average, EFW increased by 135.4 g per additional week of gestation (Table 2 and Table S1, Figure 1A). The distribution of ultrasound examinations across gestational ages was not uniform, with a higher concentration of measurements in the third trimester, reflecting routine clinical surveillance practices.

Similarly, EFW estimated using the Hadlock formula showed a strong correlation with gestational age (r = 0.86, *p* < 0.001; R^2^ = 0.728). The weekly increase in EFW was approximately 119.6 g (Table 2 and Table S2, Figure 1B).

**Figure 2:**
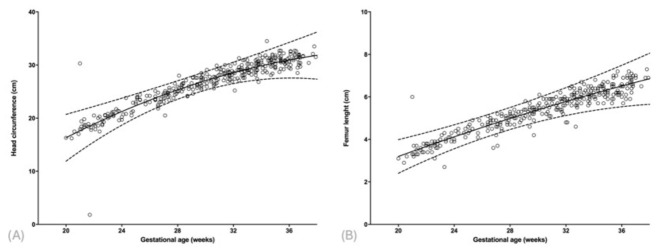
Scatter plots of head circumference (**A**) and femur length (**B**) measurements in fetuses with gastroschisis as a function of gestational age.

HC values were also strongly correlated with gestational age (r = 0.93, *p* < 0.001; R^2^ = 0.849), with a weekly mean increase of 0.83 cm (Table 2 and Table S3, Figure 2A). For FL, a significant quadratic relationship was found (r = 0.92, *p* < 0.001; R^2^ = 0.877), corresponding to a weekly growth of approximately 0.20 cm (Table 2 and Table S4, Figure 2B).

### 3.3. Comparison with Normative Growth Charts

**Figure 3:**
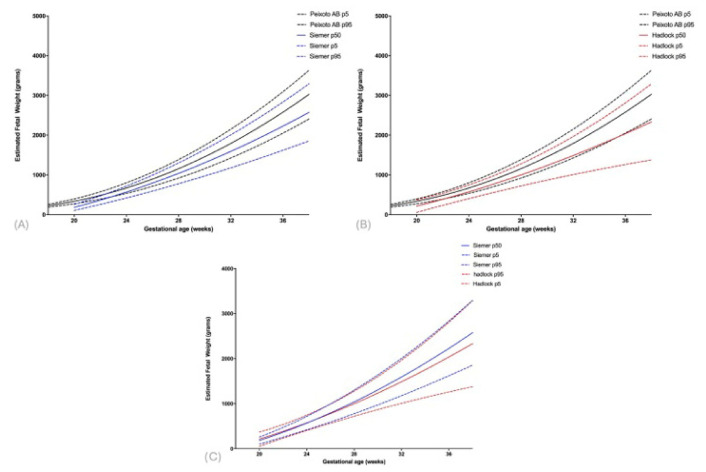
Comparison of estimated fetal weight (EFW) in fetuses with gastroschisis to the reference population, according to gestational age and the 5th, 50th, and 95th percentiles, using the Siemer formula (**A**), the Hadlock formula (**B**), and both formulas (**C**).

Comparative analyses between gastroschisis-specific and reference population curves revealed that variations in EFW were larger in gastroschisis fetuses compared to the reference population. The 50th percentile for EFW (Siemer and Hadlock) was consistently shifted to the right in fetuses with gastroschisis (Table S5; Figure 3A,B). Between 20 and 24 weeks, the median curve derived from the Siemer formula was higher than that from the Hadlock formula; however, after 24 weeks, the opposite pattern was observed (Figure 3C).

**Figure 4:**
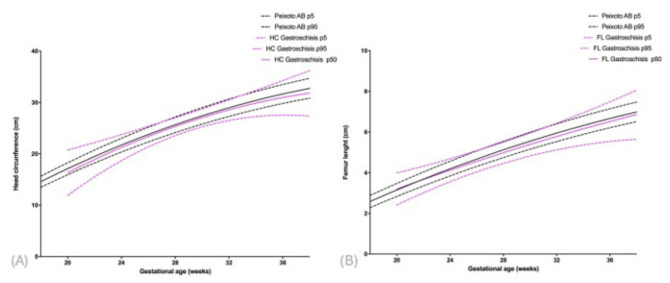
Comparison of head circumference (**A**) and femur length (**B**) measurements in fetuses with gastroschisis to the reference population, according to gestational age and the 5th, 50th, and 95th percentiles.

The 50th percentile for HC was also elevated compared to standard fetal growth charts (Table S6, Figure 4A). For FL, this rightward deviation became apparent after 24 weeks of gestation (Table S7, Figure 4B).

### 3.4. Agreement Between EFW and Birth Weight

Of the 116 cases, only 46 had both an ultrasound examination within 14 days prior to delivery and complete birthweight data, meeting the criteria for inclusion in the agreement analysis.

When using the Siemer formula, agreement with birth weight was low (CCC = 0.55; 95% CI: 0.35–0.75), and the mean absolute difference was –105.9 g. For the Hadlock formula, concordance was even lower (CCC = 0.44; 95% CI: 0.23–0.65), with a mean difference of –259.4 g (Table 3). Both models showed a statistically significant lack of agreement when the ultrasound was performed within 7 days of delivery (*p* < 0.001).

**Figure 5:**
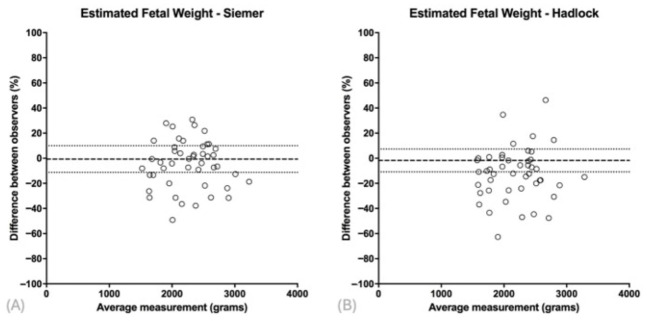
Bland–Altman plots comparing estimated fetal weight (EFW) and actual birth weight in fetuses with gastroschisis, using the Siemer formula (**A**) and the Hadlock formula (**B**).

Relative differences further demonstrated weak clinical agreement. For Siemer-derived estimates, the mean relative difference was −4.6%, with 95% limits of agreement ranging from −42.0% to +32.8% (Figure 5A). For Hadlock, the mean relative difference was −12.2%, with 95% limits from −52.8% to +28.4% (Figure 5B).

## 4. Discussion

This multicenter study shows that fetal biometry and EFW are unreliable parameters for guiding management in fetuses with gastroschisis. Although growth curves differed from standard references, the most relevant finding is the large measurement error in EFW, which can range from approximately −40% to +30%. This degree of inaccuracy, together with the low agreement between prenatal estimates and actual birth weight, limits the clinical usefulness of these measurements for decision-making in this population. Taken together, these findings indicate that estimated fetal weight should not be considered a reliable standalone parameter for clinical decision-making in gastroschisis but rather interpreted cautiously within a multimodal assessment.

Previous studies have consistently shown that fetuses with gastroschisis exhibit a symmetric reduction in all major biometric parameters and EFW when compared to standard fetal growth charts [5,15,16]. Our findings corroborate this pattern, with lower values for HC, FL, and EFW throughout gestation, suggesting a global reduction in fetal size rather than isolated abdominal growth impairment. However, unlike fetuses with growth restriction, those with gastroschisis maintain a consistent pattern of weight gain throughout gestation [15]. In the present study, no deceleration of the growth curve was identified on serial assessments, supporting the hypothesis that these fetuses may be constitutionally small rather than growth-restricted due to placental insufficiency; however, this interpretation should be made with caution, given the absence of systematic Doppler assessment, placental evaluation, and detailed neonatal outcome correlation in the present study. Notably, while AC typically shows the greatest deviation, the decrease is not restricted to this parameter [5,15,17]. The literature indicates that this growth deficit emerges as early as the second trimester and tends to persist or worsen as pregnancy progresses, with no evidence of catch-up growth [4]. The growth pattern observed is generally constitutional rather than pathological, and a high proportion of these fetuses are born small for gestational age [6,18,19]. These findings reinforce the concept that gastroschisis is associated with a distinct and stable growth trajectory, which is not adequately represented by standard population-based references.

The accuracy of fetal weight estimation in gastroschisis has been a subject of debate, particularly due to the anatomical distortion of the abdominal wall. Despite this, most studies demonstrate that standard formulas—especially those developed by Hadlock—maintain good correlation with birth weight in this population, with error rates comparable to those observed in low-risk pregnancies [6,19,20,21,22,23]. Adams et al. [24] added 30 g/day to improve the accuracy of birthweight estimation using the Hadlock III and IV formulas; however, we did not adopt this strategy, as our findings demonstrate that weight gain in fetuses with gastroschisis does not parallel that of normal fetuses.

Our results align partially with previous literature; however, in our cohort, both the Hadlock and Siemer formulas demonstrated limited agreement with birth weight, with wide limits of agreement and clinically relevant bias. These findings indicate that their accuracy is insufficient for reliable individual-level estimation, particularly when used to guide clinical decision-making. Notably, the Hadlock formula, which includes AC, tends to slightly overestimate the incidence of small for gestational age, whereas the Siemer formula—designed to minimize this bias by excluding abdominal circumference—may improve specificity but shows marginally lower precision overall [25].

These findings suggest that while both models can be applied in clinical practice, their inherent limitations should be considered when interpreting fetal size in gastroschisis, especially in the context of delivery planning and surveillance for fetal growth restriction. Furthermore, we propose that prospective studies using the Siemer formula in conjunction with condition-specific growth curves may better establish its accuracy for predicting birth weight and clarify the true clinical value of tailored reference standards in this population.

From a clinical perspective, our findings suggest that fetal biometry and estimated fetal weight should not be used in isolation to guide management decisions in pregnancies complicated by gastroschisis. The systematic deviation from standard growth charts, combined with substantial measurement error, may lead to overdiagnosis of fetal growth restriction and potentially unnecessary interventions, including iatrogenic preterm delivery. Instead, clinicians should interpret biometric parameters within the context of condition-specific growth patterns, such as those proposed in this study, and prioritize longitudinal assessment rather than single measurements. In this setting, stable growth trajectories, rather than absolute percentiles, may be more informative for surveillance, while additional parameters, such as Doppler when available, should be integrated to improve risk stratification and support individualized clinical decision-making.

From a practical standpoint, our findings have direct implications for clinical management. The systematic underestimation of fetal weight and wide limits of agreement observed in this study indicate that reliance on estimated fetal weight alone may lead to misclassification of fetal growth status and potentially unnecessary interventions, including preterm delivery. The use of condition-specific growth curves may help clinicians better distinguish constitutional smallness from true fetal growth restriction, reducing overtreatment. Furthermore, longitudinal assessment of growth trends, rather than isolated measurements, appears to be more informative in this population and should be prioritized in clinical practice.

The consistent downward shift in biometric and weight measures across gestation underscores the rationale for developing and adopting condition-specific fetal growth charts. Several studies have validated such customized references for gastroschisis, showing that they better reflect the expected, non-pathological growth pattern typical of these fetuses [5,16,18,25]. Standard population-based nomograms may inflate the perceived incidence of growth restriction, potentially prompting premature intervention and undue parental concern. In contrast, tailored curves—and, when available, individualized growth assessment tools—offer more accurate risk stratification by distinguishing physiological smallness from true intrauterine growth compromise [4]. Our findings reinforce the clinical value of integrating gastroschisis-specific growth standards into prenatal monitoring, aiming to optimize decision-making and perinatal outcomes.

The strengths of this study include its multicenter design involving tertiary referral centers across different regions of Brazil, enhancing the generalizability of the findings to diverse clinical settings. The use of standardized inclusion criteria, rigorous biometric assessment protocols, and longitudinal data collection allowed for the development of robust and clinically relevant gestational age-specific curves tailored to fetuses with gastroschisis. Additionally, the comparative analysis with established growth standards and the dual use of different weight estimation formulas provide a comprehensive evaluation of fetal growth patterns in this high-risk population.

This study has limitations inherent to its retrospective design, including potential variability in ultrasound equipment, operator technique, and data completeness across participating centers. Given that ultrasound is inherently operator-dependent and that different institutions may employ distinct imaging protocols, equipment, and surveillance strategies, these factors may have introduced unmeasured heterogeneity into the pooled dataset. Importantly, although all eligible cases from the four tertiary referral centers were included, the inability to individually link cases to their originating center precluded statistical adjustment for center-specific effects, including operator dependency and protocol variation. Therefore, despite its multicenter origin, the dataset should be interpreted as a pooled cohort rather than a fully adjusted multicenter analysis. Future prospective studies should incorporate structured center-level data collection to better control for these variables, improve internal validity, and reduce potential institutional bias. Importantly, although all eligible cases from the four tertiary referral centers were included, the final sample size was relatively small (*n* = 116). In addition, the distribution of ultrasound examinations across gestational ages was not uniform, with a higher concentration in the third trimester, reflecting routine clinical surveillance practices. The number of cases with ultrasound assessment close to delivery was also limited, which may have reduced the robustness of the comparison between EFW and birth weight. Furthermore, the interval of up to 14 days between the last ultrasound examination and delivery may have affected the accuracy of comparisons between estimated fetal weight and birthweight, potentially contributing to measurement discrepancies. Moreover, Doppler parameters were not consistently available across all participating centers due to the retrospective and multicenter design of the study, which limited the standardization of these measurements and might have introduced selection bias and reduced the ability to comprehensively assess fetal hemodynamic status across the cohort. Although the multicenter design provides a more robust and heterogeneous sample, the inability to individually link cases to their originating center made statistical adjustment for center effects unfeasible. Future research should include a structured collection of these data to allow such analyses, aiming to improve internal validity and control for potential biases. In addition, the inclusion criteria restricted to live births with at least two ultrasound examinations and absence of major anomalies or maternal disease may have introduced selection bias by excluding more severe or early complicated cases, including intrauterine deaths and cases with incomplete follow-up. This may have resulted in a cohort with relatively more stable growth patterns and potentially underestimated the variability and severity of growth alterations in gastroschisis, thereby limiting the generalizability of our findings to more severe or early-onset cases. Although the number of ultrasound examinations was sufficient to support robust curve modeling, the sample size may have limited statistical power for agreement estimates and reduced the precision of these findings. Finally, despite efforts to standardize follow-up, inter-institutional differences in clinical management may have influenced the timing and frequency of assessments.

## 5. Conclusions

In conclusion, fetuses with gastroschisis exhibit distinct growth profiles that differ from standard fetal growth charts, supporting the concept of condition-specific growth patterns in this population. The limited agreement between estimated fetal weight and actual birth weight further highlights the challenges of accurately assessing fetal size using conventional methods. These findings suggest that gastroschisis- and gestational age-specific curves may provide a more appropriate framework for interpreting fetal growth; however, further prospective studies are needed to determine their impact on clinical decision-making and perinatal outcomes.

## Figures and Tables

**Table 1:** Clinical and obstetrical ultrasound characteristics of the study population.

Variable	Value (*n* = 116)
Maternal age (years)	21.0 (19.0–38.0)
Body mass index (kg/m^2^)	24.1 (4.1)
Number of pregnancies	1.0 (1.0–9.0)
Number of deliveries	2.0 (1.0–2.0)
Gestational age at first US evaluation (weeks)	25.3 (20.0–34.8)
Biparietal diameter (cm)	6.1 (4.1–9.0)
Occipitofrontal diameter (cm)	8.3 (1.5)
Head circumference (cm)	22.7 (1.8–31.0)
Abdominal circumference (cm)	19.7 (4.1)
Femur length (cm)	4.5 (2.7–6.7)
Amniotic fluid index (cm)	11.9 (8.8–20.5)
Estimated fetal weight (Siemer) (g)	705.3 (192.3–2441.0)
Estimated fetal weight (Hadlock IV) (g)	712.8 (92.1–2329.0)
Gestational age at delivery (weeks)	37.3 (24.8–40.1)
Birth weight (g)	2434.0 (559.1)
Mode of delivery	
Vaginal	14.9% (13/87)
Cesarean section	85.1% (74/87)
Sex	
Male	45.1% (37/82)
Female	54.9% (45/82)
APGAR score at 1 min	8.0 (1.0–9.0)
APGAR score at 5 min	9.0 (3.0–10.0)

US: ultrasound. Values are presented as mean ± standard deviation or median (range), as appropriate.

**Table 2:** Influence of gestational age on estimated fetal weight by the Siemer and Hadlock formulas, head circumference, and femur length.

Variable	P5 Equation	P50 Equation	P95 Equation	r	R^2^	*p*-Value
Siemer EFW (g)	Y = 98.57 × GA − 1955	Y = 135.4 × GA − 2698	Y = 172.2 × GA − 3441	0.91	0.837	<0.0001
Hadlock EFW (g)	Y = 72.51 × GA − 1330	Y = 119.6 × GA − 2309	Y = 166.7 × GA − 3289	0.86	0.728	<0.0001
Head circumference (cm)	Y = 0.8041 × GA − 0.2119	Y = 0.8388 × GA + 1.286	Y = 0.8674 × GA + 2.891	0.93	0.849	<0.0001
Femur length (cm)	Y = 0.1733 × GA − 0.548	Y = 0.2014 × GA − 0.6989	Y = 0.2294 × GA − 0.8426	0.92	0.877	<0.0001

GA, gestational age (weeks); EFW, estimated fetal weight; r, Spearman correlation coefficient; R^2^, coefficient of determination. Linear regression model. *p* < 0.05 was considered statistically significant.

**Table 3:** Concordance correlation coefficient (CCC) values and their 95% confidence intervals (CIs) for the reproducibility of estimated fetal weight (EFW) using the Siemer and Hadlock formulas compared to birth weight.

Formula	CCC	SE	95% CI	Relative Difference (Bias/LoA) (%)	Absolute Difference (Bias/LoA) (g)
Siemer formula	0.55	0.10	0.35–0.75	−4.6/−42.0 to 32.8	−105.9/−949.5 to 737.7
Hadlock formula	0.44	0.11	0.23–0.65	−12.2/−52.8 to 28.4	−259.4/−1184.6 to 665.2

## Data Availability

The data presented in this study are available upon request from the corresponding author.

## Supplementary Materials

The following supporting information can be downloaded at: https://www.mdpi.com/article/10.3390/diagnostics16091402/s1, Table S1. Estimated fetal weight (EFW) in fetuses with gastroschisis, calculated using the Siemer formula, by gestational age and percentiles (P) 5, 50, and 95. Table S2. Estimated fetal weight (EFW) in fetuses with gastroschisis, calculated using the Hadlock formula, by gestational age and percentiles (P) 5, 50, and 95. Table S3. Head circumference (HC) in fetuses with gastroschisis, by gestational age and percentiles (P) 5, 50, and 95. Table S4. Femur length (FL) in fetuses with gastroschisis, by gestational age and percentiles (P) 5, 50, and 95. Table S5. Comparison of estimated fetal weight (EFW, in grams) between normal fetuses from the reference population and fetuses with gastroschisis, calculated using the Siemer and Hadlock formulas, by gestational age and percentiles (P) 5, 50, and 95. Table S6. Comparison of head circumference (HC, in cm) between normal fetuses from the reference population (Peixoto et al) and fetuses with gastroschisis, by gestational age and percentiles 5, 50, and 95. Table S7. Comparison of femur length (FL, in cm) between normal fetuses from the reference population and fetuses with gastroschisis, by gestational age and percentiles (P) 5, 50, and 95.
