# Supplementary material for: Gestational Age-Specific Biometric and Estimated Fetal Weight Curves in Gastroschisis: A Brazilian Multicenter Cohort Study
Source: Diagnostics (Basel). 2026 May 6;16(9):1402. doi: 10.3390/diagnostics16091402 (PMC13163731; doi:10.3390/diagnostics16091402)
Supplement: Supplementary file 1 [file diagnostics-16-01402-s001.zip › diagnostics-4260408-supplementary.pdf]

SUPPLEMENTAL MATERIAL

Table S1. Estimated fetal weight (EFW) in fetuses with gastroschisis, calculated using the Siemer formula, by gestational age and percentiles (P) 5, 50, and 95.

Gestational age (weeks)	Total (N)	P5 (g)	P50 (g)	P95 (g)
20	4	103	180	257
21	14	178	269	360
22	16	256	364	472
23	14	336	463	591
24	9	419	568	717
25	11	505	678	851
26	14	593	793	993
27	22	684	913	1143
28	19	777	1039	1300
29	26	873	1169	1465
30	21	972	1305	1638
31	27	1073	1446	1818
32	26	1177	1592	2006
33	32	1284	1743	2201
34	28	1393	1899	2405
35	30	1505	2060	2615
36	31	1619	2227	2834
37	7	1736	2398	3060
38	4	1856	2575	3294

Table S2. Estimated fetal weight (EFW) in fetuses with gastroschisis, calculated using the Hadlock formula, by gestational age and percentiles (P) 5, 50, and 95.

Gestational age (weeks)	Total (N)	P5 (g)	P50 (g)	P95 (g)
20	4	54	212	369
21	14	145	297	449
22	16	233	385	538
23	14	319	478	636
24	9	403	574	745
25	11	486	674	863
26	14	566	779	991
27	22	645	887	1129
28	19	721	999	1277
29	26	795	1115	1434
30	21	868	1235	1601
31	27	938	1358	1778
32	26	1007	1486	1965
33	32	1073	1617	2162
34	28	1138	1753	2368
35	30	1200	1892	2584
36	31	1261	2035	2810
37	7	1319	2182	3045
38	4	1376	2333	3291

Table S3. Head circumference (HC) in fetuses with gastroschisis, by gestational age and percentiles (P) 5, 50, and 95.

Gestational age (weeks)	Total (N)	P5 (cm)	P50 (cm)	P95 (cm)
20	4	11.9	16.3	20.7
21	14	13.8	17.6	21.4
22	16	15.5	18.9	22.2
23	14	17.2	20.1	23.0
24	9	18.7	21.2	23.8
25	11	20.1	22.3	24.5
26	14	21.4	23.4	25.4
27	22	22.5	24.4	26.2
28	19	23.5	25.3	27.0
29	26	24.5	26.2	27.9
30	21	25.3	27.1	28.7
31	27	25.9	27.8	29.6
32	26	26.5	28.6	30.5
33	32	26.9	29.2	31.4
34	28	27.3	29.9	32.4
35	30	27.5	30.4	33.3
36	31	27.5	31.0	34.3
37	7	27.5	31.4	35.2
38	4	27.3	31.8	36.2

Table S4. Femur length (FL) in fetuses with gastroschisis, by gestational age and percentiles (P) 5, 50, and 95.

Gestational age (weeks)	Total (N)	P5 (cm)	P50 (cm)	P95 (cm)
20	4	2.4	3.2	4.0
21	14	2.7	3.4	4.2
22	16	3.0	3.7	4.3
23	14	3.3	3.9	4.5
24	9	3.6	4.1	4.7
25	11	3.8	4.3	4.9
26	14	4.0	4.6	5.1
27	22	4.3	4.8	5.3
28	19	4.5	5.0	5.5
29	26	4.7	5.2	5.7
30	21	4.8	5.4	6.0
31	27	5.0	5.6	6.2
32	26	5.1	5.8	6.4
33	32	5.3	6.0	6.7
34	28	5.4	6.2	6.9
35	30	5.5	6.3	7.2
36	31	5.5	6.5	7.5
37	7	5.6	6.7	7.8
38	4	5.7	6.9	8.1

Table S5. Comparison of estimated fetal weight (EFW, in grams) between normal fetuses from the reference population and fetuses with gastroschisis, calculated using the Siemer and Hadlock formulas, by gestational age and percentiles (P) 5, 50, and 95.

GA (weeks)	Peixoto	Siemer	Hadlock	Peixoto	Siemer	Hadlock	Peixoto	Siemer	Hadlock
		P5			P50			P95	
20	273	103	54	336	180	212	395	257	369
21	328	178	145	406	269	297	479	360	449
22	390	256	233	485	364	385	575	472	538
23	459	336	319	573	463	478	682	591	636
24	537	419	403	671	568	574	800	717	745
25	621	505	486	779	678	674	930	851	863
26	714	593	566	895	793	779	1070	993	991
27	813	684	645	1021	913	887	1222	1143	1129
28	921	777	721	1157	1039	999	1385	1300	1277
29	1036	873	795	1302	1169	1115	1559	1465	1434
30	1158	972	868	1456	1305	1235	1745	1638	1601
31	1288	1073	938	1620	1446	1358	1942	1818	1778
32	1425	1177	1007	1793	1592	1486	2150	2006	1965
33	1571	1284	1073	1975	1743	1617	2369	2201	2162
34	1723	1393	1138	2167	1899	1753	2600	2405	2368
35	1883	1505	1200	2369	2060	1892	2842	2615	2584
36	2051	1619	1261	2579	2227	2035	3095	2834	2810
37	2226	1736	1319	2799	2398	2182	3359	3060	3045
38	2409	1856	1376	3029	2575	2333	3634	3294	3291

GA: gestational age

Table S6. Comparison of head circumference (HC, in cm) between normal fetuses from the reference population (Peixoto et al) and fetuses with gastroschisis, by gestational age and percentiles 5, 50, and 95.

	Normal	Gastroschisis	Normal	Gastroschisis	Normal	Gastroschisis
**GA (weeks)**	**P5**		**P50**		**P95**	
20	15.9	11.9	17.1	16.3	18.3	20.7
21	17.1	13.8	18.3	17.6	19.5	21.4
22	18.2	15.5	19.5	18.9	20.7	22.2
23	19.3	17.2	20.6	20.1	21.9	23.0
24	20.4	18.7	21.7	21.2	23.0	23.8
25	21.4	20.1	22.7	22.3	24.1	24.5
26	22.3	21.4	23.8	23.4	25.2	25.4
27	23.2	22.5	24.7	24.4	26.2	26.2
28	24.1	23.5	25.7	25.3	27.2	27.0
29	25.0	24.5	26.5	26.2	28.1	27.9
30	25.8	25.3	27.4	27.1	29.0	28.7
31	26.6	25.9	28.2	27.8	29.8	29.6
32	27.3	26.5	29.0	28.6	30.7	30.5
33	28.0	26.9	29.7	29.2	31.4	31.4
34	28.6	27.3	30.4	29.9	32.2	32.4
35	29.2	27.5	31.0	30.4	32.8	33.3
36	29.8	27.5	31.7	31.0	33.5	34.3
37	30.3	27.5	32.2	31.4	34.1	35.2
38	30.8	27.3	32.8	31.8	34.7	36.2

GA: gestational age

Table S7. Comparison of femur length (FL, in cm) between normal fetuses from the reference population and fetuses with gastroschisis, by gestational age and percentiles (P) 5, 50, and 95.

	**Normal**	**Gastroschisis**	**Normal**	**Gastroschisis**	**Normal**	**Gastroschisis**
**GA (weeks)**	**P5**		**P50**		**P95**	
20	2.8	2.4	3.1	3.2	3.5	4.0
21	3.1	2.7	3.4	3.4	3.7	4.2
22	3.4	3.0	3.7	3.7	4.0	4.3
23	3.6	3.3	3.9	3.9	4.3	4.5
24	3.8	3.6	4.2	4.1	4.6	4.7
25	4.1	3.8	4.4	4.3	4.8	4.9
26	4.3	4.0	4.7	4.6	5.1	5.1
27	4.5	4.3	4.9	4.8	5.3	5.3
28	4.7	4.5	5.1	5.0	5.5	5.5
29	5.0	4.7	5.3	5.2	5.8	5.7
30	5.2	4.8	5.6	5.4	6.0	6.0
31	5.3	5.0	5.8	5.6	6.2	6.2
32	5.5	5.1	6.0	5.8	6.4	6.4
33	5.7	5.3	6.1	6.0	6.6	6.7
34	5.9	5.4	6.3	6.2	6.8	6.9
35	6.1	5.5	6.5	6.3	7.0	7.2
36	6.2	5.5	6.7	6.5	7.1	7.5
37	6.4	5.6	6.8	6.7	7.3	7.8
38	6.5	5.7	7.0	6.9	7.5	8.1

GA: gestational age
